# The Impact of Specialised Heart Failure Outpatient Care on the Long-Term Application of Guideline-Directed Medical Therapy and on Prognosis in Heart Failure with Reduced Ejection Fraction

**DOI:** 10.3390/diagnostics14020131

**Published:** 2024-01-06

**Authors:** Balázs Muk, Fanni Bánfi-Bacsárdi, Máté Vámos, Dávid Pilecky, Zsuzsanna Majoros, Gábor Márton Török, Dénes Vágány, Balázs Polgár, Balázs Solymossi, Tünde Dóra Borsányi, Péter Andréka, Gábor Zoltán Duray, Róbert Gábor Kiss, Miklós Dékány, Noémi Nyolczas

**Affiliations:** 1 Department of Adult Cardiology, Gottsegen National Cardiovascular Center, 1096 Budapest, Hungary; 2 Cardiac Electrophysiology Division, Cardiology Center, Internal Medicine Clinic, University of Szeged, 6720 Szeged, Hungary; 3 Doctoral School of Clinical Medicine, University of Szeged, 6720 Szeged, Hungary; 4 Department of Cardiology, Central Hospital of Northern Pest—Military Hospital, 1134 Budapest, Hungary; 5 Heart and Vascular Center, Semmelweis University, 1122 Budapest, Hungary

**Keywords:** heart failure outpatient care, heart failure with reduced ejection fraction, prognosis

## Abstract

(1) Background: Besides the use of guideline-directed medical therapy (GDMT), multidisciplinary heart failure (HF) outpatient care (HFOC) is of strategic importance in HFrEF. (2) Methods: Data from 257 hospitalised HFrEF patients between 2019 and 2021 were retrospectively analysed. Application and target doses of GDMT were compared between HFOC and non-HFOC patients at discharge and at 1 year. 1-year all-cause mortality (ACM) and rehospitalisation (ACH) rates were compared using the Cox proportional hazard model. The effect of HFOC on GDMT and on prognosis after propensity score matching (PSM) of 168 patients and the independent predictors of 1-year ACM and ACH were also evaluated. (3) Results: At 1 year, the application of RASi, MRA and triple therapy (TT: RASi + βB + MRA) was higher (*p* < 0.05) in the HFOC group, as was the proportion of target doses of ARNI, βB, MRA and TT. After PSM, the composite of 1-year ACM or ACH was more favourable with HFOC (propensity-adjusted HR = 0.625, 95% CI = 0.401–0.974, *p* = 0.038). Independent predictors of 1-year ACM were age, systolic blood pressure, application of TT and HFOC, while 1-year ACH was influenced by the application of TT. (4) Conclusions: HFOC may positively impact GDMT use and prognosis in HFrEF even within the first year of its initiation.

## 1. Introduction

Heart failure (HF) is one of the leading cardiovascular diseases, with a high risk of morbidity and mortality [1,2] and a prognosis comparable to that of the most common cancers [3]. The 1-year mortality risk of HF is estimated to be 15–30%, and the 5-year risk can approach 75% in specific populations, while 50% of the patients are readmitted to hospital within 1 year after the initial diagnosis of HF [4].

In terms of heart failure with reduced ejection fraction (HFrEF), randomised clinical trials (RCTs) published in recent decades have provided strong evidence for the application of disease-modifying drug regimes; thus, current guidelines recommend the early implementation of the four pillars of HFrEF, including angiotensin-converting enzyme inhibitors (ACEi)/angiotensin receptor neprilysin inhibitors (ARNIs), beta-blockers (βB), mineralocorticoid receptor antagonists (MRAs) and the sodium–glucose cotransporter 2 inhibitor (SGLT2i) dapagliflozin/empagliflozin [5].

Besides the use of guideline-directed medical therapy (GDMT), multidisciplinary HF outpatient care (HFOC) is of strategic importance in the management and prognosis of HFrEF [5,6,7]. The current 2021 European Society of Cardiology (ESC) Guidelines for the diagnosis and the treatment of acute and chronic HF recommend enrolling all HF patients in a multidisciplinary HF management programme to reduce the risk of HF hospitalisation and mortality (class of recommendation: I, level of evidence: A) [5]. It is, however, well known that HFOC is not currently broadly established and reimbursed worldwide, despite the robust supporting evidence [8].

Studies that have suggested the importance of HFOC among HF patients were essentially small case series, and published before the paradigm shift in pharmacotherapy in HFrEF [8,9,10,11,12,13,14,15,16,17,18,19,20,21,22]. Moreover, most of these analyses were heterogeneous, did not apply a standardised protocol regarding the implementation of HFOC [9,10,14,15,16,17,18,19,20,21,22] and evaluated selected populations; in some of them, only short-term follow-up (FUP) was applied. Furthermore, it has to be highlighted that most of these analyses did not examine the impact of other underlying confounding factors or even pharmacotherapy on prognosis. Furthermore, these well-known studies that assessed the impact of HFOC typically did not investigate patient compliance.

Furthermore, we can also find publications in the literature that do not support the claim that HFOC may have a significant positive effect on prognosis, as the meta-analysis of Takeda et al. [23] suggested.

As the prevalence of HF continues to rise due to the ageing population, better diagnostic tools and improved therapeutic options [4], there is a growing need for a dedicated, specialised HFOC network. Although in 2011 the ESC Heart Failure Association [6] and in 2008 the Heart Failure Society of America [24] also published documents focusing on the key elements of HF care, HFOC does not have a broadly accepted standardised protocol [25]. It is generally accepted that its main elements involve educating, monitoring, clinically following and supporting the patient with an emphasis on self-care, regularly considering therapy optimisation possibilities and adopting a multidisciplinary, holistic approach that focuses on managing the growing number of comorbidities [5,8,25,26,27].

The ESC HF Guidelines published in 2021 and their focused update in 2023 also emphasise the crucial role of the post-discharge phase after an HF hospitalisation [5,28], as this represents a critical, vulnerable period in which outpatient care is of paramount importance [29] for preventing adverse events, death and recurrent hospitalisations.

In our retrospective observational study, we aimed to evaluate the impact of HFOC on the application rate and achieved target doses of GDMT, to analyse its effect on the composite endpoint of 1-year all-cause mortality and rehospitalisation and to investigate the independent predictors of 1-year all-cause mortality and all-cause rehospitalisation in HFrEF patients after hospitalisation for HF.

## 2. Materials and Methods

### 2.1. Study Population and Design

We undertook a retrospective observational study, analysing a consecutive, non-selected group of real-world patients with HFrEF hospitalised for HF between 1 January 2019 and 31 October 2021 in a tertiary cardiac centre, in the HF Unit of the Department of Cardiology, Medical Centre, Hungarian Defence Forces. In-hospital mortality was an exclusion criterion. In the case of multiple hospitalisations during the data collection period, the first event was considered in the analysis to avoid redundancy. Patients were followed up for 1 year. All patients were offered regular FUP at our HF Outpatient Clinic; its acceptance was voluntary. The optional decisions of the patients may have had multifactorial origins (including patient preference, socioeconomic status, distance of HF Outpatient Clinic, family support, etc.), which—due to the design of our study—were not projected to be investigated originally. “HFOC patients” refers to those who accepted FUP and had regular ambulatory visits at our HF Outpatient Clinic. Those who refused the opportunity for FUP at our HF Outpatient Clinic are referred to as “non-HFOC patients”. For HFOC patients, a structured, patient-centred and individualised follow-up was initiated in which patient management was led by a cardiologist specialising in HF, working in close collaboration with HF nurses [13,25,30] and other cardiology subspecialties and specialists of other comorbidities. For those participating in the HFOC at our centre, the schedule of outpatient visits was individualised (in general, in-office controls were undertaken every 3 months, with variable but more frequent in-office and remote controls for those with treatment optimisation in the post-discharge phase and those with more advanced stages of the disease).

Our retrospective observational study protocol was reviewed and approved by the Institutional Research Ethics Committee of the Medical Centre, Hungarian Defence Forces (approval number: KK00/144-1/2022.), and the present study adheres to the ethical principles of the Declaration of Helsinki (1975, revised in 2013). For our retrospective observational study, no written informed consent was required as our research did not influence the professional medical care of the patients, required no intervention and involved only retrospective data collection in an anonymised form.

### 2.2. Study Outcomes

In comparing HFOC and non-HFOC patients, the application of conventional neurohormonal antagonist therapy (RASi: ACEi/angiotensin receptor blocker (ARB)/ARNI, βB, MRA) was evaluated at hospital discharge and at 1 year, as well as the proportion of patients receiving target doses. 1-year prognosis (all-cause mortality, all-cause rehospitalisation, rehospitalisation for acute HF (AHF) and the composite endpoint of 1-year all-cause mortality and all-cause rehospitalisation) was investigated. The independent predictors of 1-year all-cause mortality and 1-year all-cause rehospitalisation were also examined.

### 2.3. Statistical Analysis

Clinical data were obtained from our hospital’s information system, and mortality data were acquired through the electronic social insurance number validity documentation interface of the National Health Insurance Fund. Data were documented in anonymised form in a Microsoft Excel 16.80 spreadsheet (Microsoft Corporation, Redmond, WA, USA), and statistical calculations were undertaken using IBM SPSS Statistics 26.0 (International Business Machines Corporation, Armonk, NY, USA).

The distribution of continuous variables was tested with a Shapiro–Wilk normality test. Based on their non-Gaussian distribution, continuous variables were presented as median and interquartile ranges, while categorical variables were expressed as absolute numbers and percentages. The descriptive characteristics of HFOC and non-HFOC were compared using Fisher’s exact test and the Mann–Whitney test (as applicable).

Patients followed up at our HF Outpatient Clinic were also matched in a 1:1 ratio with patients not followed up at our HF Outpatient Clinic using the nearest neighbour matching method with a calliper of 0.2 with adjustment for possible confounders at hospital discharge (female gender, age, de novo HFrEF, ischaemic aetiology, atrial fibrillation/flutter, diabetes mellitus, hypertension, systolic blood pressure, heart rate, creatinine level, potassium level, haemoglobin level, left ventricular ejection fraction (LVEF), cardiac resynchronisation therapy (CRT) at hospital discharge, RASi-, βB- and MRA medication at discharge). After propensity score matching (PSM), the 1-year application ratio of neurohormonal antagonist therapy for patients assigned to the HFOC and non-HFOC groups was also evaluated using Fisher’s exact test.

Mortality and rehospitalisation rates in the total cohort of HFOC and non-HFOC patients and afterwards among the propensity score-matched cohort were assessed using the Kaplan–Meier method and log-rank test, and they were compared using the univariate Cox proportional hazard model.

The independent predictors of 1-year all-cause mortality and rehospitalisation were investigated with uni- and multivariate Cox regression analysis for the whole cohort.

Statistical significance was defined as *p* < 0.05.

## 3. Results

### 3.1. Patient Population

A total of 257 patients were involved in our retrospective analysis. A total of 74% of them were male, and the median age was 65 [55–73] years. A total of 40% of patients required hospitalisation for HF before the index event, 32% were newly diagnosed (de novo) HFrEF patients and 45% had at least partly ischaemic aetiology of HF. Median LVEF was 25 [20–30]%. Diabetes mellitus affected 40% of the population, while hypertension affected 62% and atrial fibrillation/flutter 46%. At hospital discharge, 89% of the patients were on RASi medication (ACEi/ARB: 71%, ARNI: 18%), 85% were on βB, 95% were on MRA and altogether 77% were receiving triple therapy (RASi (ACEi/ARB/ARNI) + βB + MRA), while SGLT2i application was 11%. As for the target doses achieved at hospital discharge, 23% of patients were at target doses of RASi, 22% of βB, 68% of MRA and 6% of triple therapy. A total of 19% had cardiac resynchronisation therapy with or without defibrillator (CRT-P/CRT-D), while 22% possessed an implantable cardioverter defibrillator (ICD) without CRT. At hospital discharge, 44% of the patients accepted HFOC, while 56% rejected it for personal reasons. Table 1 shows the main characteristics of the examined patient cohort.

Comparison of the baseline characteristics of HFOC and non-HFOC patient subgroups showed significant deviation in terms of age, the proportion of de novo HFrEF patients and medical and device therapy at discharge (in terms of the ratio of CRT-P/CRT-D, RASi, triple therapy and target doses of βB) (Table 1).

After PSM (Figure S1), 84 patients each were assigned to the HFOC and non-HFOC groups, with no differences in the relevant baseline characteristics and therapy (Table S1). Regarding distance from the HFOC, no significant difference was identified between the matched groups. Based on the data from the Hungarian Central Statistical Office [31], no significant deviations were expected in their income.

Of the 257 patients, 10 were lost to FUP at 1 year. We were able to analyse 1-year pharmacotherapy in 191 patients who were alive at 1 year.

### 3.2. Impact of Heart Failure Outpatient Care on the Application of Guideline-Directed Medical Therapy and Therapy Adherence

**Figure 1:**
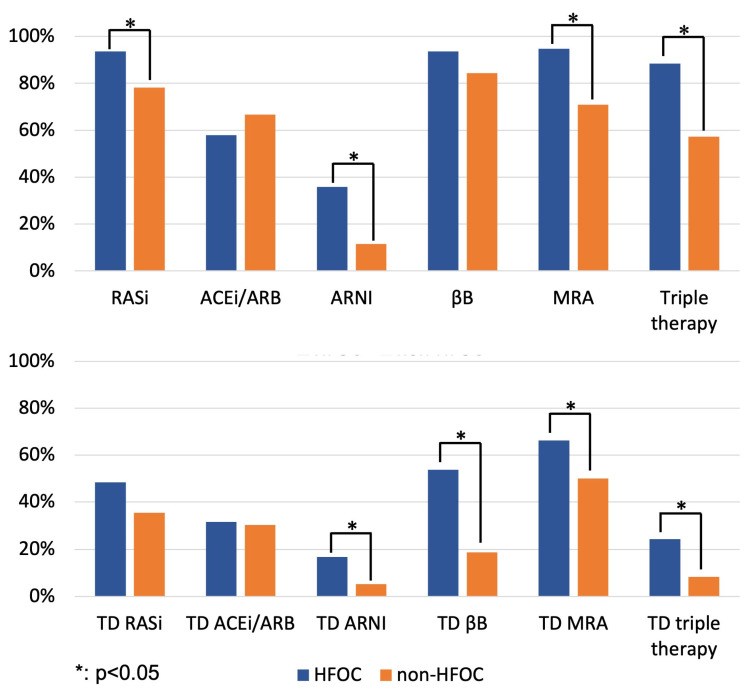
Proportion of patients on GDMT and target doses of GDMT at 1 year (comparison of HFOC and non-HFOC subgroups). ACEi: angiotensin-converting enzyme inhibitor; ARB: angiotensin receptor blocker; ARNI: angiotensin receptor neprilysin inhibitor; βB: beta-blocker; HFOC: heart failure outpatient care; MRA: mineralocorticoid receptor antagonist; RASi: renin–angiotensin system inhibitor; TD: target dose.

At 1 year, the proportion of patients on RASi (94% vs. 78%, *p* = 0.007; HFOC vs. non-HFOC group, respectively), MRA (95% vs. 71%, *p* < 0.001) and triple therapy (88% vs. 57%, *p* < 0.001) was significantly larger among those followed up with HFOC (Figure 1; Table S2). Regarding target doses, the proportion of target doses of ARNI (17% vs. 5%, *p* = 0.011), βB (54% vs. 19%, *p* < 0.001), MRA (66% vs. 50%, *p* = 0.028) and triple therapy medication (24% vs. 8%, *p* = 0.003) among HFOC patients exceeded the pertinent data for the non-HFOC group (Figure 1; Table S2).

At 1 year, neurohormonal antagonist therapy was discontinued in 2–5% of HFOC patients, significantly less (*p* < 0.001) than in the case of the 10–28% discontinuation rate of the non-HFOC group (RASi: 4% vs. 18%; βB: 2% vs. 10%; MRA: 5% vs. 23%; triple therapy: 5% vs. 28%), suggesting that HFOC had an impact on therapy adherence.

**Figure 2:**
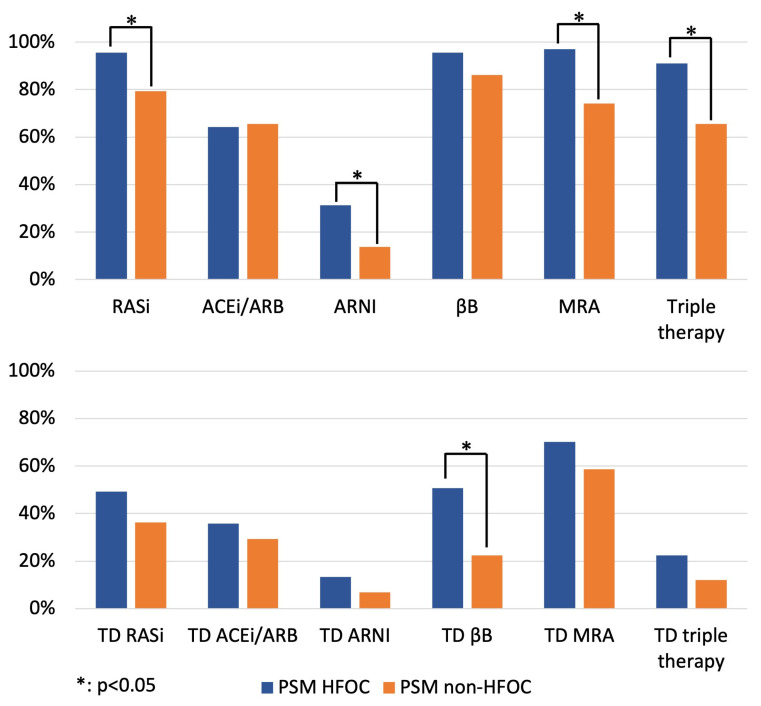
Proportion of patients on GDMT and target doses of GDMT at 1 year (comparison of HFOC and non-HFOC subgroups after PSM). ACEi: angiotensin-converting enzyme inhibitor; ARB: angiotensin receptor blocker; ARNI: angiotensin receptor neprilysin inhibitor; βB: beta-blocker; HFOC: heart failure outpatient care; MRA: mineralocorticoid receptor antagonist; PSM: propensity score matching; RASi: renin–angiotensin system inhibitor; TD: target dose.

After PSM, the application ratios of RASi (96% vs. 79%, *p* = 0.011), MRA (97% vs. 74%, *p* < 0.001) and triple therapy (91% vs. 66%, *p* < 0.001) remained higher in the HFOC group (Figure 2; Table S2), while management in HFOC was accompanied by a significantly higher application ratio of target doses of βBs (51% vs. 22%, *p* = 0.002) (Figure 2; Table S2) and resulted in favourable trends in terms of the proportion of patients on target doses of RASi-s, MRAs and triple therapy at 1 year.

### 3.3. Heart Failure Outpatient Care and the Prognosis of HFrEF

**Figure 3:**
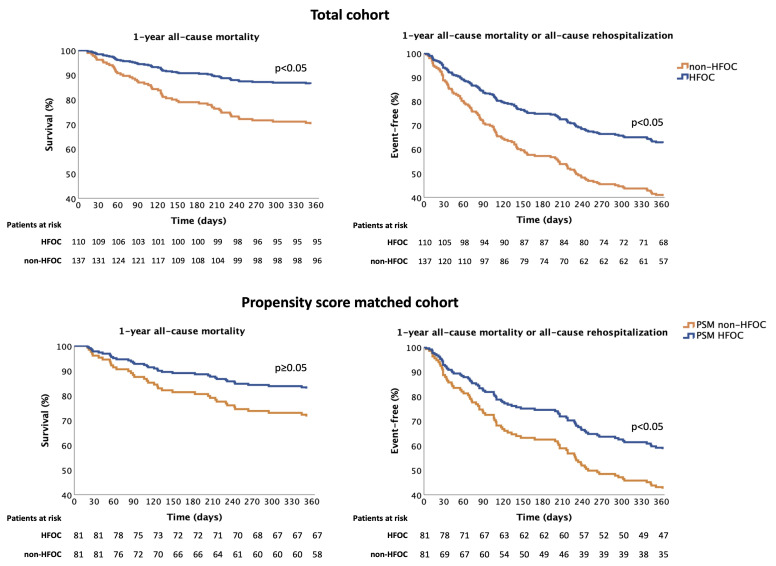
Effect of HFOC on the composite endpoint of 1-year all-cause mortality and all-cause rehospitalisation. HFOC: heart failure outpatient care; PSM: propensity score matching.

1-year all-cause mortality was 23% in the whole cohort. All-cause rehospitalisation affected 39%, and rehospitalisation for AHF occurred in 17%. Comparison of the 1-year prognosis in HFOC and non-HFOC subgroups shows that the all-cause mortality rate for patients followed up with HFOC was significantly lower (14% vs. 30%, HR = 0.412, 95% confidence interval (CI) = 0.228–0.744, *p* = 0.003), with fewer all-cause rehospitalisations (34% vs. 43%, HR = 0.619, 95% CI = 0.410–0.934, *p* = 0.022); consequently, the composite endpoints of all-cause mortality or all-cause rehospitalisation rates were also more favourable (38% vs. 58%, HR = 0.520, 95% CI = 0.358–0.756, *p* = 0.001). Rehospitalisation for AHF also showed a favourable trend in the case of HFOC management (14% vs. 20%, HR = 0.566, 95% CI = 0.302–1.061, *p* = 0.076) (Table 2, Figure 3).

Analysis of data from 168 patients after PSM (1:1 matching) shows that the composite endpoint of 1-year all-cause mortality or all-cause rehospitalisation was significantly more favourable in the case of HFOC, leading to a 37.5% relative-risk reduction (propensity-adjusted HR = 0.625, 95% CI = 0.401–0.974, *p* = 0.038) (Figure 3). When the composite endpoint elements were examined separately, with regard to the effect of HFOC, nonsignificant favourable trends were observed in 1-year all-cause mortality (propensity-adjusted HR = 0.563, 95% CI = 0.290–1.095, *p* = 0.090). In contrast, the frequency of 1-year all-cause rehospitalisation did not differ (propensity-adjusted HR = 0.744, 95% CI = 0.455–1.216, *p* = 0.238). No significant deviation was seen in terms of the 1-year rehospitalisation for AHF (propensity-adjusted HR = 0.522, 95% CI = 0.255–1.068, *p* = 0.075). The results of univariate Cox regression analyses are shown in Table 3.

### 3.4. Independent Predictors of 1-Year All-Cause Mortality and All-Cause Rehospitalisation

The predictors of 1-year all-cause mortality and all-cause rehospitalisation confirmed using univariate Cox regression analysis are presented in Table S3. In the multivariate Cox regression model, younger age (adjusted HR = 1.039, 95% confidence interval (CI) = 1.009–1.070, *p* = 0.010), higher systolic blood pressure (adjusted HR = 0.983, 95% CI = 0.970–0.997, *p* = 0.017), HFOC (adjusted HR = 0.501, 95% CI = 0.269–0.933, *p* = 0.029) and the application of triple therapy (adjusted HR = 0.528, 95% CI = 0.289–0.965, *p* = 0.038) favourably influenced 1-year all-cause mortality (Table 4).

Regarding the predictors of 1-year all-cause rehospitalisation, in the multivariate Cox regression model, triple therapy application (adjusted HR = 0.572, 95% CI = 0.358–0.912, *p* = 0.019) significantly reduced the risk of all-cause rehospitalisation at 1 year, while follow-up with HFOC was associated with a favourable trend (adjusted HR = 0.696, 95% CI = 0.453–1.068, *p* = 0.097) (Table 4).

## 4. Discussion

### 4.1. Main Findings

Applying HFOC proved to be an independent predictor of 1-year all-cause mortality for the whole cohort based on multivariate Cox regression analysis.

After the correction for the correctable potential confounders using propensity score matching, HFOC was associated with a significant positive impact on the prognosis of HFrEF patients even within the first 1 year of its initiation, reducing the composite endpoint of all-cause mortality and all-cause rehospitalisation at 1 year by 37.5%.

The implementation of the complex drug therapy of HFrEF and the proportion of target doses of the neurohormonal antagonist therapy that were achieved were significantly more favourable in the group of patients who were followed up with HFOC, and the discontinuation rate of GDMT was lower among the participants of the HFOC, suggesting the essential role of HFOC in therapy adherence.

### 4.2. Impact of Heart Failure Outpatient Care on the Application of Guideline-Directed Medical Therapy and Therapy Adherence

Even though the need for hospitalisation is an unfavourable prognostic marker of HF [32], the opportunity to initiate disease-modifying drug therapy and continue up-titration to target doses [33,34] must be taken, as time-to-GDMT is a modifiable risk factor of prognosis in HFrEF [35,36]. The COACH trial revealed that patients with higher mortality risk profited from longer in-hospital therapy optimisation, leading to a significant reduction in all-cause death or cardiovascular hospitalisation [37]. Based on the results of the STRONG-HF trial [38], which confirmed the importance of predischarge and early post-discharge phase intensive care after acute HF hospitalisation, the Focused Update of the 2021 ESC Guidelines for HF recommends for all patients the initiation and rapid up-titration of evidence-based treatment before discharge and during frequent and careful follow-up visits within the first 6 weeks following an HF hospitalisation, in order to reduce the risk of HF rehospitalisation or death [28].

According to the results of recently published data and our analysis, HFOC greatly impacts the implementation and continuous optimisation of novel GDMT [38,39]. A recently published cohort study by Dunlay et al. revealed that care at an HF clinic is independently associated with the initiation of new first-line HFrEF drugs (referring to all conventional neurohormonal antagonist therapies) among de novo HFrEF patients, leading to a 1.54–2.49-fold increase in their implementation [39]. In our patient cohort, HFOC resulted in a significantly higher application rate of triple therapy at 1 year, and the number of patients at target doses of triple therapy also exceeded that in the non-HFOC group.

The proportion of patients on GDMT was similar to that described in the ESC Heart Failure Long-Term Registry [40] and the Hungarian HF Registry [41]; however, the proportion of comorbidities was significantly higher in our cohort. The importance of the implementation of the first-line HFrEF drug regime cannot be underscored enough, although even in recently published RCTs and registries that examined different therapeutical modalities in HFrEF, the proportion of patients on triple therapy and at target doses of triple therapy was remarkably small [42,43,44], highlighting the importance of awareness of the continuous need for therapy optimisation.

In the STRONG-HF trial, which assessed the safety and efficacy of ”high-intensity care” among HF patients with non-fully optimised treatment, “high-intensity care” was safe and led to the notably higher application rate of target doses of GDMT (RASi: 55% vs. 2%, βB: 49% vs. 4%, MRA: 84% vs. 46%; high-intensity care vs. usual care group) [38]. However, one should keep in mind that in the STRONG-HF trial, only those patients could be randomised for whom eGFR ≥ 30 mL/min/1.73 m^2^, NT-proBNP at screening >2500 pg/mL and at randomisation >1500 pg/mL with a >10% decrease between screening and before randomisation, serum potassium level ≤ 5.0 mmol/L, systolic blood pressure ≥ 100 mmHg and heart rate ≥ 60 min^−1^ [38].

In contrast, the HFrEF patient population in our retrospective observational study, with a larger burden of comorbidities and more advanced stages of dysfunction, may represent the everyday practice of HFOC better. Despite this, the proportion of patients receiving target doses of neurohormonal antagonist therapy followed up at our HF Outpatient Clinic (RASi: 48%, βB: 54%, MRA: 66%, triple therapy: 24%) approached the results of the “high-intensity care” care group in the STRONG-HF trial [38] and exceeded the target dose application rate of first-line therapy in the VICTORIA registry (RASi: 13.8%, ARNI: 19.9%, βB: 17.5%, MRA: 71.2%, triple therapy: 1.4%) [44].

Even though a significant difference was observed between the HFOC and non-HFOC groups in terms of the target doses of GDMT in our analysis, a notable proportion of non-HFOC patients were on target doses of these disease-modifying drugs as well at 1 year of FUP (RASi: 35%, βB: 19%, MRA: 50%), underscoring—besides the importance of the intrahospital implementation and optimisation of GDMT [33]—the essential role of primary care in maintaining the already initiated and up-titrated GDMT of HFrEF [45]. HFOC has a profound effect not only on the implementation of GDMT but also on its long-term application and on therapy adherence, as the EVOLUTION HF study confirmed that GDMT might be discontinued in 23.5–42.2% of patients considering the four pillars of HFrEF treatment within a year after initiation [46]. According to our analyses, the discontinuation rate of triple therapy was more than five times as high if patients were not followed up with HFOC. The meta-analysis of Jonkman et al. also confirmed the importance of the length of therapeutic interventions, finding that each additional month of intervention can reduce the risk of mortality and HF-related hospitalisation by 1–4% [26].

### 4.3. Heart Failure Outpatient Care and the Prognosis of HFrEF

While the application of quadruple therapy (ARNI + βB + MRA + SGLT2i) reduces the risk of all-cause mortality in HFrEF patients by 61%, and the use of triple therapy (ACEi/ARNI + βB + MRA) by 48–56% [47], the multidisciplinary care of HF may reduce mortality by 25% [48]. According to our analysis, proper HFOC can reduce the composite endpoint of all-cause mortality and all-cause rehospitalisation by 37.5%, even at 1 year.

The interpretation of the data in the literature regarding the efficacy of HFOC on patients’ prognosis is often controversial, leaning on the meta-analyses of small trials/RCTs. In 2014, the meta-analysis of Feltner et al., which included 47 trials, revealed that multidisciplinary HF clinics reduced all-cause readmission and mortality rates [49]. On the other hand, based on the meta-analysis of 47 RCTs, Takada et al. found limited evidence for the effect of disease management programmes on HF mortality [23], potentially suggesting the superiority of case management on all-cause mortality/HF readmission reduction compared with clinic-based interventions.

The efficacy of HFOC may be greater during the early post-discharge period after HF hospitalisation [29]. According to Koser et al., HFOC after hospital discharge reduced 30-day hospital readmission (13.3% vs. 22%; HFOC vs. national average in the USA) as well as 30-day mortality rates (1.2% vs. 11.6%) [50]. The STRONG-HF trial clearly showed that close FUP after AHF hospitalisation notably reduced the composite endpoint of all-cause death and HF readmission even within 180 days (15.2% vs. 23.3%; high-intensity care vs. usual care group). According to another publication, HFOC may reduce further HF hospitalisations by 26% and all-cause hospitalisations by 19% [48]. Recently, Van Spall et al. concluded that after HF hospitalisation, FUP at disease management clinics reduced all-cause mortality by 20% [8].

Even though it is recommended that HF patients are followed up at HF outpatient clinics [6], the optimal duration of HFOC programmes has not been established yet. Previously, a few studies attempted to identify whether patients benefit from HFOC after therapy optimisation or can be safely managed with primary care. In 2013, the NorthStar study showed no benefit of HFOC compared with primary care in HFrEF patients already receiving GDMT, even in the high-risk population (NT-proBNP ≥ 1000 pg/mL) [51]. In accordance, the COACH-2 study [52] also revealed no difference in the number of deaths and hospital readmissions for cardiovascular causes when comparing HFOC versus primary care in clinically stable patients, with actual GDMT based on ESC guidelines from 2008. Moreover, the extended follow-up of the NorthStar trial revealed that long-term, 10-year FUP at specialised HF clinics did not reduce the composite endpoint of HF hospitalisation or cardiovascular death compared with FUP at primary care [45]. Despite this, it must be highlighted that these studies referred to stable patients already receiving GDMT and showing no signs of worsening heart failure. Moreover, the former were published before the results of the latest RCTs which have reformed the complex pharmacotherapy of HFrEF, and it is a well-known fact that HF outpatient care has a huge impact on the implementation and long-term application of novel GDMT [38,39]. Based on the disease’s progressive nature [53], the regular revision of drug and device options and the need for advanced HF therapies is essential. These results may indicate that the proper, individualised selection of HF patient populations requiring special care at HF outpatient clinics is needed to achieve the best outcomes.

### 4.4. Independent Predictors of 1-Year All-Cause Mortality and All-Cause Rehospitalisation

HFOC independently reduced the risk of 1-year all-cause mortality in the total cohort in our study, in addition to the beneficial, 1-year mortality-reducing effects of younger age, higher systolic blood pressure and the application of triple therapy. Implementing multidisciplinary HFOC was already proven to be independently associated with reduced risk of total mortality and all-cause hospital readmissions in the ICONS registry in 2009 [54], and its beneficial effect on mortality has remained stable in recent years as well [55]. In agreement with our results, the ESC Heart Failure Long-Term Registry and the international cohort study by Lam et al. also revealed age and blood pressure to be parameters significantly influencing mortality [40,56]. Triple therapy application not only influenced mortality but also independently reduced the risk of all-cause rehospitalisation at 1 year in our study, in accordance with the largest RCTs and international data [57,58].

## 5. Conclusions

Besides implementing a disease-modifying drug regime, FUP with a multidisciplinary HF programme is strategically important for reducing morbidity and mortality in HFrEF patients. According to the results of our study, which assessed the data of a real-world HFrEF patient cohort requiring hospitalisation due to HF, HFOC may cause significant improvement in terms of prognosis, even within the first year of its initiation. Moreover, our results highlight that HFOC positively affects the application rate of GDMT and the proportion of achieved target doses, resulting in better long-term therapy adherence. Therefore, one should insist on the implementation of HFOC in everyday practice.

### Limitations

The patient population of our single-centre study consisted exclusively of individuals of the Caucasian race, so our results and conclusions cannot be applied with certainty to those outside this group. A further limitation is the small study cohort. However, the latter approximated the patient populations of the RCTs published in recent decades that evaluated the effect of HFOC in patients hospitalised for HF [8]. Participation in HFOC was voluntary, and patients’ decisions may have had multifactorial origins (including patient preference, socioeconomic status, distance of the HF Outpatient Clinic, family support, etc.); however, not all of these factors could be investigated in our study. The voluntary participation of patients in the HFOC might impact the results. Current funding regulations may have negatively influenced the rate of ARNI use in Hungary as well. As the results of the SGLT2i landmark trials (DAPA-HF [59], EMPEROR-REDUCED [60]) were published during the time of our study and were only incorporated into the 2021 ESC HF guidelines [5], the proportion of patients on dapagliflozin and empagliflozin was not assessed in the present study. HFOC in Hungary is not fully reimbursed; thus, the network of HFOC services is not well established.

## Figures and Tables

**Table 1:** Main baseline characteristics of the study population.

Parameters	Total Cohort (*n* = 257)	HFOC (*n* = 114)	Non-HFOC (*n* = 143)	*p*-Value
Male gender, *n* (%)	191 (74)	88 (77)	103 (72)	0.390
Age, median [IQR, years	65 [55–73]	64 [52–69]	67 [58–76]	0.001
Previous hospitalisation primarily due to heart failure, *n* (%)	103 (40)	59 (52)	44 (31)	0.001
De novo HFrEF, *n* (%)	83 (32)	25 (22)	58 (41)	0.002
Ischaemic aetiology, *n* (%)	115 (45)	45 (39)	70 (49)	0.133
LVEF, median [IQR], %	25 [20–30]	25 [20–30]	25 [20–30]	0.934
Heart rate, median [IQR], min^−1^	88 [74–100]	83 [69–100]	90 [75–102]	0.099
Systolic blood pressure, median [IQR], mmHg	117 [102–134]	115 [100–131]	120 [104–135]	0.098
Distance from HFOC, median [IQR]), km	9 [5–68]	17 [6–76]	5 [8–36]	0.025
Comorbidities				
Diabetes, *n* (%)	104 (40)	42 (37)	62 (43)	0.308
Hypertension, *n* (%)	159 (62)	66 (58)	93 (65)	0.248
Atrial fibrillation/flutter, *n* (%)	118 (46)	57 (50)	61 (43)	0.259
Laboratory parameters at hospital discharge				
Creatinine, median [IQR], μmol/L	111 [87–146]	111 [89–142]	111 [86–147]	0.993
eGFR, median [IQR], mL/min/1.73 m^2^	58 [39–75]	58 [39–76]	58 [38–71]	0.378
Potassium, median [IQR], mmol/L	4.4 [4.0–4.7]	4.4 [4.1–4.7]	4.3 [4.0–4.7]	0.530
Haemoglobin, median [IQR], g/L	121 [106–138]	123 [109–138]	120 [104–138]	0.539
NT-proBNP, median [IQR], pg/mL	6492 [3296–12,000]	8404 [4283–14,161]	5453 [2785–9655]	0.001
Medical and device therapy at hospital discharge				
RASi, *n* (%)	228 (89)	107 (94)	121 (85)	0.028
ACEi/ARB, *n* (%)	183 (71)	74 (65)	109 (76)	0.053
ARNI, *n* (%)	45 (18)	33 (29)	12 (9)	<0.001
βB, *n* (%)	219 (85)	102 (89)	117 (82)	0.111
MRA, *n* (%)	244 (95)	111 (97)	133 (93)	0.154
Triple therapy, *n* (%)	198 (77)	95 (83)	103 (72)	0.037
SGLT2i, *n* (%)	28 (11)	17 (15)	11 (8)	0.072
TD RASi, *n* (%)	58 (23)	27 (24)	31 (22)	0.764
TD ACEi/ARB, *n* (%)	48 (19)	20 (18)	28 (20)	0.748
TD ARNI, *n* (%)	10 (4)	7 (6)	3 (2)	0.114
TD βB, *n* (%)	57 (22)	38 (33)	19 (13)	<0.001
TD MRA, *n* (%)	175 (68)	77 (68)	98 (69)	0.893
TD triple therapy, *n* (%)	16 (6)	8 (7)	8 (6)	0.796
CRT-P/CRT-D, *n* (%)	48 (19)	34 (30)	14 (10)	<0.001
ICD, *n* (%)	56 (22)	31 (27)	25 (17)	0.069

ACEi: angiotensin-converting enzyme inhibitor; ARB: angiotensin receptor blocker; ARNI: angiotensin receptor neprilysin inhibitor; βB: beta-blocker; CRT-P/CRT-D: cardiac resynchronisation therapy pacemaker/defibrillator; eGFR: estimated glomerular filtration rate; HFOC: heart failure outpatient care; HFrEF: heart failure with reduced ejection fraction; ICD: implantable cardioverter defibrillator; [IQR]: interquartile range; LVEF: left ventricular ejection fraction; MRA: mineralocorticoid receptor antagonist; NT-proBNP: N-terminal pro-B type natriuretic peptide; RASi: renin–angiotensin system inhibitor; SGLT2i: sodium–glucose cotransporter 2 inhibitor; TD: target dose.

**Table 2:** 1-year prognosis—comparison of HFOC and non-HFOC patients: results of Cox regression analyses.

Prognosis at 1 Year	HFOC (*n* = 110)	Non-HFOC (*n* = 137)	Univariate Cox Regression Analysis			
			HR	95% CI		*p*-Value
All-cause mortality	15 (14)	41 (30)	0.412	0.228	0.744	0.003
All-cause rehospitalisation	37 (34)	59 (43)	0.619	0.410	0.934	0.022
Rehospitalisation for AHF	15 (14)	28 (20)	0.566	0.302	1.061	0.076
All-cause mortality and all-cause rehospitalisation	42 (38)	80 (58)	0.520	0.358	0.756	0.001

AHF: acute heart failure; CI: confidence interval; HFOC: heart failure outpatient care; HR: hazard ratio.

**Table 3:** 1-year prognosis in PSM groups of HFOC and non-HFOC patients: results of Cox regression analyses.

Prognosis at 1 Year	HFOC (*n* = 81)	Non-HFOC (*n* = 81)	Univariate Cox Regression Analysis			
			HR	95% CI		*p*-Value
All-cause mortality	14 (17)	23 (28)	0.563	0.290	1.095	0.090
All-cause rehospitalisation	30 (37)	34 (42)	0.744	0.455	1.216	0.238
Rehospitalisation for AHF	12 (15)	20 (25)	0.522	0.255	1.068	0.075
All-cause mortality and all-cause rehospitalisation	34 (42)	46 (57)	0.625	0.401	0.974	0.038

AHF: acute heart failure; CI: confidence interval; HFOC: heart failure outpatient care; HR: hazard ratio; PSM: propensity score matching.

**Table 4:** Independent predictors of 1-year all-cause mortality and 1-year all-cause rehospitalisation using multivariate Cox regression analysis.

**1-Year All-Cause Mortality**				
	**Adjusted HR**	**95% CI**		***p*** **-Value**
**Age (/1 year)**	**1.039**	**1.009**	**1.070**	**0.010**
**Systolic blood pressure (/1 mmHg)**	**0.983**	**0.970**	**0.997**	**0.017**
eGFR at discharge (/1 mL/min/1.73 m^2^)	0.993	0.978	1.008	0.329
Diabetes	1.271	0.724	2.231	0.404
Atrial fibrillation/flutter	1.546	0.866	2.761	0.141
**HFOC**	**0.501**	**0.269**	**0.933**	**0.029**
**Triple therapy at discharge**	**0.528**	**0.289**	**0.965**	**0.038**
**1-Year All-Cause Rehospitalisation**				
	**Adjusted HR**	**95% CI**		***p*** **-Value**
Age (/1 year)	1.009	0.990	1.029	0.339
eGFR at discharge (/1 mL/min/1.73 m^2^)	0.996	0.985	1.007	0.427
Diabetes	1.244	0.824	1.876	0.298
Atrial fibrillation/flutter	1.382	0.910	2.099	0.129
HFOC	0.696	0.453	1.068	0.097
**Triple therapy at discharge**	**0.572**	**0.358**	**0.912**	**0.019**

CI: confidence interval; eGFR: estimated glomerular filtration rate; HFOC: heart failure outpatient care; HR: hazard ratio. The independent predictors proven are marked as bold.

## Data Availability

All data generated or analysed during this study are included in this article. Further enquiries can be directed to the corresponding author.

## Supplementary Materials

The following supporting information can be downloaded at https://www.mdpi.com/article/10.3390/diagnostics14020131/s1: Supplementary Materials are provided in the text, with the following legends: Figure S1: Dot plot of propensity score matching. Table S1: Main baseline characteristics of the study population after propensity score matching. Table S2: Medical and device therapy at 1 year in the total cohort and after propensity score matching. Table S3: Predictors of 1-year all-cause mortality and 1-year all-cause rehospitalisation using univariate Cox regression analysis.
