# Supplementary material for: The Impact of Specialised Heart Failure Outpatient Care on the Long-Term Application of Guideline-Directed Medical Therapy and on Prognosis in Heart Failure with Reduced Ejection Fraction
Source: Diagnostics (Basel). 2024 Jan 6;14(2):131. doi: 10.3390/diagnostics14020131 (PMC10814730; doi:10.3390/diagnostics14020131)
Supplement: Supplementary file 1 [file diagnostics-14-00131-s001.zip › diagnostics-2716515-supplementary.pdf]

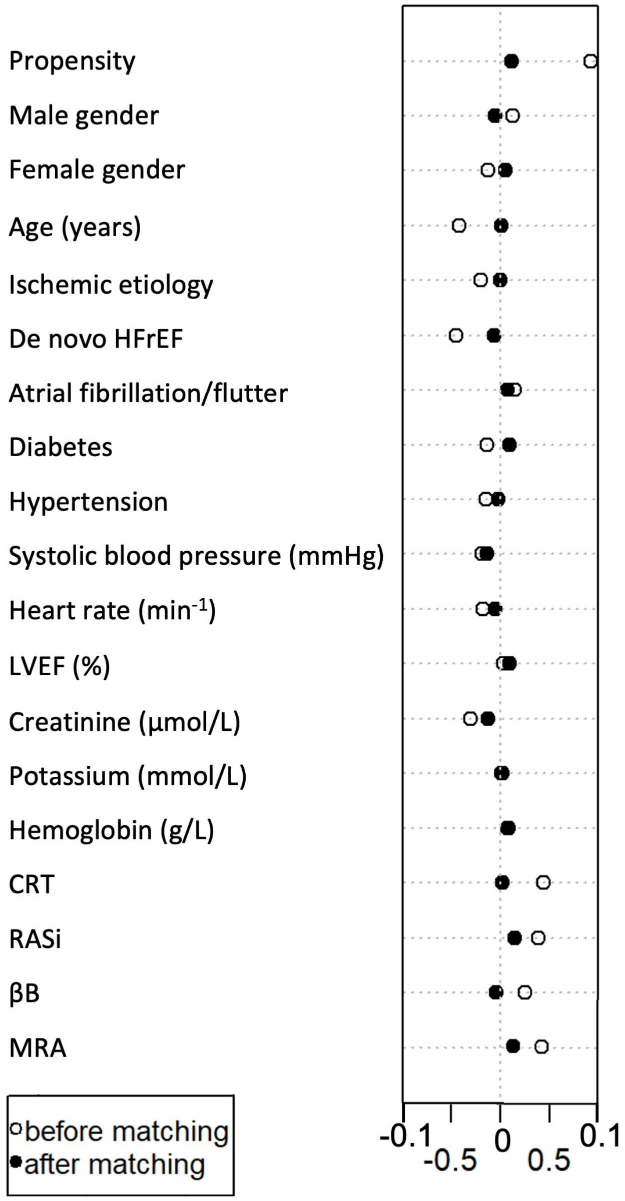

Figure S1. Dot plot of propensity-score matching.

βB: beta-blocker; CRT: cardiac resynchronization therapy; HFrEF: heart failure with reduced ejection fraction; LVEF: left ventricular ejection fraction; MRA: mineralocorticoid receptor antagonist; RASi: renin-angiotensin system inhibitor.

Table S1. Main baseline characteristics of the study population after propensity-score matching.

Parameters	PSM total cohort (*n* = 168)	PSM HFOC (*n* = 84)	PSM non-HFOC (*n* = 84)	*p*-Value
Male gender, *n* (%)	132 (79)	65 (77)	67 (80)	0.851
Age, median [IQR], years	63 [50-69]	64 [52-70]	62 [48-68]	0.623
Previous hospitalization primarily due to heart failure, n (%)	70 (42)	37 (44)	33 (39)	0.639
De novo HfrEF, *n* (%)	48 (29)	23 (27)	25 (30)	0.865
Ischemic etiology, *n* (%)	74 (44)	37 (44)	37 (44)	1.000
LVEF, median [IQR], %	25 [20-30]	25 [20-30]	25 [19-30]	0.611
Heart rate, median [IQR], min^{-1}	87 [71-100]	85 [70-100]	88 [73-100]	0.495
Systolic blood pressure, median [IQR], mmHg	118 [102-137]	117 [102-137]	120 [103-135]	0.586
Distance from HFOC, median [IQR], km	11 [5-67]	13 [5-73]	8 [6-54]	0.578
**Comorbidities**				
Diabetes, *n* (%)	66 (39)	35 (42)	31 (37)	0.636
Hypertension, *n* (%)	107 (64)	53 (63)	54 (64)	1.000
Atrial fibrillation/flutter, *n* (%)	69 (41)	36 (43)	33 (38)	0.754
**Laboratory parameters at hospital discharge**				
creatinine, median [IQR], μmol/L	111 [85-147]	112 [86-155]	106 [83-145]	0.499
eGFR, median [IQR], $mL/min/1.73m^2$	61 [39-83]	58 [38-79]	63 [42-86]	0.423
potassium, median [IQR], mmol/L	4.4 [4.1-4.7]	4.4 [4.1-4.7]	4.4 [4.1-4.7]	0.975
hemoglobin, median [IQR], g/L	122 [108-138]	124 [108-139]	121 [106-138]	0.685
**Medical and device therapy at hospital discharge**				
RASi, *n* (%)	153 (91)	78 (93)	75 (89)	0.590
ACEi/ARB, *n* (%)	123 (73)	58 (69)	65 (77)	0.296
ARNI, *n* (%)	30 (18)	20 (24)	10 (12)	0.069
βB, *n* (%)	149 (89)	74 (88)	75 (89)	1.000
MRA, *n* (%)	160 (95)	81 (96)	79 (94)	0.720
Triple therapy, *n* (%)	136 (81)	68 (81)	68 (81)	1.000
SGLT2i, *n* (%)	21 (13)	12 (14)	9 (11)	0.642
TD RASi, *n* (%)	43 (26)	19 (23)	24 (29)	0.480
TD ACEi/ARB, *n* (%)	37 (22)	16 (19)	21 (25)	0.457
TD ARNI, *n* (%)	6 (4)	3 (4)	3 (4)	1.000
TD βB, *n* (%)	36 (21)	23 (27)	13 (15)	0.090
TD MRA, *n* (%)	120 (71)	58 (69)	62 (74)	0.609
TD Triple therapy, *n* (%)	11 (7)	5 (6)	6 (7)	1.000
CRT-P/CRT-D, *n* (%)	27 (16)	14 (17)	13 (15)	1.000
ICD, *n* (%)	48 (29)	27 (32)	21 (25)	0.393

ACEi: angiotensin-converting enzyme inhibitor; ARB: angiotensin receptor blocker; ARNI: angiotensin receptor neprilysin inhibitor; βB: beta-blocker; CRT-P/CRT-D: cardiac resynchronization therapy pacemaker/defibrillator; eGFR: estimated glomerular filtration rate; HFOC: heart failure outpatient care; HfrEF: heart failure with reduced ejection fraction; ICD: implantable cardioverter defibrillator; [IQR]: interquartile range; LVEF: left ventricular ejection fraction; MRA: mineralocorticoid receptor antagonist; PSM: propensity-score matching; RASi: renin-angiotensin system inhibitor; SGLT2i**:** sodium-glucose cotransporter 2 inhibitor; TD: target dose.

Table S2. Medical and device therapy at 1 year in the total cohort and after propensity-score matching.

	**Total cohort (*n* = 191)**	**HFOC (*n* = 95)**	**non-HFOC (*n* = 96)**	***p*-Value**	**PSM total cohort (*n* = 125)**	**PSM HFOC (*n* = 67)**	**PSM non-HFOC (*n* = 58)**	***p*-Value**
RASi, *n* (%)	163 (85)	89 (94)	75 (78)	0.007	110 (88)	64 (96)	46 (79)	0.011
ACEi/ARB, *n* (%)	118 (61)	55 (58)	64 (67)	0.182	81 (65)	43 (64)	38 (65)	1.000
ARNI, *n* (%)	45 (24)	34 (36)	11 (11)	<0.001	29 (23)	21 (32)	8 (14)	0.033
βB, *n* (%)	170 (89)	89 (94)	81 (84)	0.062	114 (91)	64 (96)	50 (86)	0.111
MRA, *n* (%)	158 (83)	90 (95)	68 (71)	<0.001	108 (86)	65 (97)	43 (74)	<0.001
Triple therapy, *n* (%)	139 (73)	84 (88)	55 (57)	<0.001	99 (79)	61 (91)	38 (66)	<0.001
SGLT2i, *n* (%)	25 (13)	17 (18)	8 (8)	0.056	17 (14)	11 (16)	6 (10)	0.434
TD RASi, *n* (%)	80 (42)	46 (48)	34 (35)	0.079	54 (43)	33 (49)	21 (36)	0.153
TD ACEi/ARB, *n* (%)	59 (31)	30 (31)	29 (30)	0.876	41 (33)	24 (36)	17 (29)	0.453
TD ARNI, *n* (%)	21 (11)	16 (17)	5 (5)	0.011	13 (10)	9 (13)	4 (7)	0.258
TD βB, *n* (%)	69 (36)	51 (54)	18 (19)	<0.001	47 (38)	34 (51)	13 (22)	0.002
TD MRA, *n* (%)	111 (58)	63 (66)	48 (50)	0.028	81 (65)	47 (70)	34 (59)	0.194
TD Triple therapy, *n* (%)	31 (16)	23 (24)	8 (8)	0.003	22 (18)	15 (22)	7 (12)	0.161
CRT-P/CRT-D, *n* (%)	39 (20)	30 (32)	9 (9)	<0.001	18 (14)	11 (16)	7 (12)	0.612
ICD, *n* (%)	44 (23)	27 (28)	17 (18)	0.088	36 (29)	23 (34)	13 (22)	0.168

ACEi: angiotensin-converting enzyme inhibitor; ARB: angiotensin receptor blocker; ARNI: angiotensin receptor neprilysin inhibitor; βB: beta-blocker; CRT-P/CRT-D: cardiac resynchronization therapy pacemaker/defibrillator; eGFR: estimated glomerular filtration rate; HFOC: heart failure outpatient care; ICD: implantable cardioverter defibrillator; LVEF: left ventricular ejection fraction; MRA: mineralocorticoid receptor antagonist; PSM: propensity-score matching; RASi: renin-angiotensin system inhibitor; SGLT2i: sodium-glucose cotransporter 2 inhibitor; TD: target dose.

Table S3. Predictors of 1-year all-cause mortality and 1-year all-cause rehospitalization using univariate Cox regression analysis.

**1-year all-cause mortality**				
	**HR**	**95% CI**		***p*-Value**
**Age (/1 year)**	**1.058**	**1.034**	**1.083**	**<0.001**
Female gender	1.084	0.600	1.959	0.789
Heart rate (/1 min^{-1})	0.992	0.980	1.004	0.215
**Systolic blood pressure (/1 mmHg)**	**0.983**	**0.971**	**0.996**	**0.012**
**eGFR at discharge (/1 mL/min/1.73 m^2)**	**0.975**	**0.963**	**0.987**	**<0.001**
Potassium at admission > 4.5 mmol/L	1.068	0.632	1.805	0.807
Left ventricular ejection fraction (/1%)	0.991	0.953	1.030	0.648
**Diabetes**	**1.721**	**1.019**	**2.908**	**0.042**
Hypertension	1.485	0.832	2.653	0.181
**Atrial fibrillation/flutter**	**1.986**	**1.162**	**3.397**	**0.012**
Coronary artery disease	0.976	0.577	1.650	0.928
CRT at discharge	1.101	0.570	2.219	0.775
**HFOC**	**0.412**	**0.228**	**0.744**	**0.003**
**Triple therapy at discharge**	**0.286**	**0.169**	**0.484**	**<0.001**
**1-year all-cause rehospitalization**				
	**HR**	**95% CI**		***p*-Value**
**Age (/1 year)**	**1.025**	**1.009**	**1.041**	**0.002**
Female gender	0.677	0.409	1.118	0.127
Heart rate (/1 min^{-1})	1.001	0.992	1.009	0.899
Systolic blood pressure (/1 mmHg)	0.999	0.990	1.008	0.784
**eGFR at discharge (/1 mL/min/1.73 m^2)**	**0.988**	**0.979**	**0.998**	**0.013**
Potassium at discharge > 4.5 mmol/L	0.790	0.526	1.187	0.257
Left ventricular ejection fraction (/1%)	0.992	0.964	1.021	0.603
**Diabetes**	**1.438**	**0.961**	**2.151**	**0.077**
Hypertension	1.223	0.800	1.869	0.354
**Atrial fibrillation/flutter**	**1.405**	**0.942**	**2.098**	**0.096**
Coronary artery disease	1.347	0.902	2.011	0.146
CRT at discharge	0.840	0.484	1.457	0.534
**HFOC**	**0.619**	**0.410**	**0.934**	**0.022**
**Triple therapy at discharge**	**0.463**	**0.300**	**0.715**	**0.001**

CI: confidence interval; CRT: cardiac resynchronization therapy; eGFR: estimated glomerular filtration rate; HFOC: heart failure outpatient care; HR: hazard ratio. The predictors proven are marked as bold.
